# Current Challenges in the Bioinformatics of Single Cell Genomics

**DOI:** 10.3389/fonc.2014.00007

**Published:** 2014-01-27

**Authors:** Luwen Ning, Geng Liu, Guibo Li, Yong Hou, Yin Tong, Jiankui He

**Affiliations:** ^1^Department of Biology, South University of Science and Technology of China, Shenzhen, China; ^2^BGI-Shenzhen, Shenzhen, China

**Keywords:** single cell analysis, RNA sequencing, DNA sequencing, SNP, CNV

## Abstract

Single cell genomics is a rapidly growing field with many new techniques emerging in the past few years. However, few bioinformatics tools specific for single cell genomics analysis are available. Single cell DNA/RNA sequencing data usually have low genome coverage and high amplification bias, which makes bioinformatics analysis challenging. Many current bioinformatics tools developed for bulk cell sequencing do not work well with single cell sequencing data. Here, we summarize current challenges in the bioinformatics analysis of single cell genomic DNA sequencing and single cell transcriptomes. These challenges include calling copy number variations, identifying mutated genes in tumor samples, reconstructing cell lineages, recovering low abundant transcripts, and improving the accuracy of quantitative analysis of transcripts. Development in single cell genomics bioinformatics analysis will promote the application of this technology to basic biology and medical research.

## Introduction

Biologists have been interested in the heterogeneity between individual single cells at the molecular level in tissues and organs for a long time. For example, what is the difference between adjacent cells at the genetic and gene expression level in a tumor? What is different between cells at different developmental stages of human embryogenesis? These questions, and many similar questions, remain largely unanswered.

Novel sequencing technologies have rapidly advanced genomics studies in the past few years. Now, there are several exciting new techniques that enable us to sequence entire genomes at the single cell level. For example, multiple displacement amplification (MDA) has widely been used to amplify entire genomes from a few cells or even a single cell (1–3). Zong et al. recently described a multiple annealing, looping-based amplification cycle (MALBAC) method, which combines features of linear amplification methods with PCR (4). MALBAC has been shown to be capable of amplifying 93% of the genome of a single cell. Single cell sequencing technology has potentially broad applications in biology and medicine (5–7); for example, in the characterization of the earliest differentiation events in human embryogenesis (8); in the study of microorganisms that cannot be cultured (9–12); in transcriptome analysis of rare, circulating tumor cells (13–15); and in the study of tumor heterogeneity and microevolution (16–19).

The difference between single cell sequencing and bulk sequencing is that single cell sequencing needs an extra step that amplifies the genome from a single cell. It is this amplification process that makes the bioinformatics analysis of single cell sequencing data so challenging. The amplification process has two major technical problems. First, single cell amplification usually has a much lower genome coverage. Genomic regions that are not amplified will not be sequenced. Second, the amplification process will introduce artificial biases, with some genomic regions being amplified more than others. Because of these two reasons, many bioinformatics tools developed for bulk cell sequencing do not work well for single cell sequencing data. Nevertheless, as a revolutionary technology, single cell sequencing will quickly be applied in many biological and medical fields, and the bioinformatics community needs to act quickly to keep pace with the expected flood of single cell sequencing data.

In this review, we will describe the challenges in analyzing single cell DNA and RNA sequencing data. In addition, we will discuss the comparative analysis of multiple single cells.

## Section I: Bioinformatics in Single Cell DNA Sequencing

Single-nucleotide polymorphisms (SNPs) contribute most of the genetic variation to the human genome (20). SNPs associate with many monogenic and complex diseases, such as cancer, autoimmune disorders, diabetes, and Alzheimer’s (21–24). Copy number variation (CNV) is another major type of genetic polymorphism (25) that has important roles in human health (26). CNV has been reported to be associated with various human diseases, such as tumors, autism, autoimmunity, systematic lupus erythematous, and other complex diseases (27–30). Analyzing DNA mutation and structural variation at the single cell level has been reported in a few recent studies (4, 31, 32). However, accurately calling SNP/CNV from single cell sequencing data remains challenging.

## SNP Calling in Single Cell DNA Sequencing

Calling SNPs in single cell data is a challenge that stems from the whole genome amplification (WGA) process itself. Typically, there are only about 6 pg of DNA in a single cell, and therefore, accurately measuring all of DNA information content from within such a small amount is very difficult. WGA from single cells is an essential step during the library preparation for high throughput sequencing. Although the first WGA techniques appeared more than 10 years ago (33), current WGA methods still suffer from low coverage performance and errors during amplification, which present a hurdle in obtaining complete SNP information from a single cell.

### Low genome coverage causes SNP dropout

Genome coverage is a measurement of the percentage of a genome covered by at least, one sequencing read. Current WGA techniques often have a lower genome coverage than bulk cell sequencing. For example, the MDA method can typically obtain an average genome coverage of 73% at a 25× sequencing depth (4). The recently developed MALBAC method enables significant improvements over traditional MDA methods, and can reach a genome coverage of 93% at a 30× sequencing depth (with an average genome coverage of 34% at a 25× sequencing depth) (4). The genome coverage of single cell amplification methods is still much lower than that of bulk cell sequencing, which can achieve more than 90% coverage at a 4× sequencing depth. SNPs in genomic regions that are not covered by sequencing reads will drop out of the analysis owing to these reasons. Furthermore, MDA methods suffer from a high ratio of allele dropout (ADO, alleles present in heterozygous samples not called out in the analysis). The ADO rate of MDA methods can be as high as 65%, as estimated by Zong et al. (4) Therefore, we should be very careful using single cell SNP calling results to perform further analyses such as those for gene ontology and pathway enrichment studies.

### Errors in amplification lead to false-positive SNP calling

Although WGA methods usually use a high fidelity polymerase enzyme, single cell sequencing still introduces a certain amount of false-positive error in calling SNPs. The MDA methods use Φ29 bacteriophage DNA polymerase, which has been shown to have a low error rate, approximately 10^−5^ per base (35). Zong et al. sequenced DNA from single cells amplified with both MALBAC and MDA methods, and found that the false-positive rate for genotyping single-nucleotide variants with MALBAC was about 40-fold higher than it was for MDA (36). They identified 2.2 × 10^6^ SNPs in a single cell using GATK software (37), but the data contained 1.1 × 10^5^ false positives, which means that one in 20 SNPs is artificial.

### Strategies for developing single cell SNP calling algorithms

Single-nucleotide polymorphisms calling algorithms for bulk cell samples have been studied extensively; among them, GATK (37), SNPdetector (38), SOAPsnp (39), and VarScan (40) are widely used. However, there is no SNP calling algorithm originally designed for single cell sequencing data. Researchers have used established software to call SNPs in a few recently published single cell studies. For example, Zong et al. (4) used GATK, while Xu et al. (19) used SOAPsnp to call SNPs in single cell sequencing samples. None of these methods, however, take the intrinsic properties of single cell amplification into consideration. To develop a SNP calling algorithm specifically designed for single cell sequencing, and to overcome the low genome coverage and high false-positive rate shortcomings, we have the following two suggestions: (1) the algorithm should be able to distinguish true SNPs from amplification errors; and (2) the algorithm should be able to call SNPs from low coverage sequencing.

## CNV Calling in Single Cell Sequencing

Copy number variation in genomes results in cells having an abnormal number of copies of one or more sections of DNA. Currently many software packages are available for calling CNVs in bulk DNA sequencing, such as CNV-seq (41), PenCNV (42), CNAseg (43), Readdepth (44), and cn.MOPS (45). However, few software packages and algorithms have been designed for single cell CNV calling, and the impact of amplification bias on CNV calling has not been systematically investigated.

### Amplification bias in single cell CNV calling

Multiple displacement amplification bias has been observed in several studies (46). For example, MDA has been reported to introduce hundreds of potentially confounding CNV artifacts that can obscure the detection of real variants (47). Many artifacts are reproducible, and may correlate with proximity to chromosome ends and GC content. The WGA4 (Sigma Genomeplex) method and MALBAC also have strong read fluctuations mapped to different genome regions. The majority of CNV calling methods in bulk cells are read count-based approaches. As an example, Readdepth is reported to be able to detect CNVs with sizes as small as 500 bp in 37× sequencing depths (44). However, in single cell sequencing the bin size must be increased to reduce read mapping bias when using read count-based approaches, owing to strong amplification biases. Navin et al. adopted a variable length bins method with a medium length of 54 kb (18). In MALBAC, the CNVs of single cell samples were analyzed at a resolution of 200 kb bin size (4). Baslan et al. proposed a protocol to analyze the CNV of single cell sequencing using a default bin size of 50 kb (48). The reproducibility of single cell CNV detection is also relatively low. Zong et al. (4) reported that the read number in 200 kb bins of two single cells from the same tissue has a correlation coefficient value less than 0.8.

### Strategies for accurate single cell CNV calling

To improve the quality and accuracy of calling CNV from single cell sequencing, we have the following suggestions: First, we need to carefully examine the bias generated in the genome amplification process. For example, in one study of the MDA method, recurrent MDA-induced copy number biases were reported to associate with sequence repeats and proximity to chromosome ends, increased GC content, and annotated CNVs (47). Once we know the pattern of artificial biases, we can develop algorithms to reduce the noise to call confident CNV assessment. Second, noise reduction problems also exist in other fields, such as signal processing and image processing, where noise reduction has been extensively studied. We can employ algorithms such as wavelet (49) and/or Fourier transformation (50) to single cell data to reduce noise. Third, pairwise comparisons of amplified products should help to reduce the number of artificial CNVs.

## Section II: Single cell RNA sequencing

The identity and function of a cell is determined by its entire RNA component. Ideally, transcriptome analysis should capture the exact quantity of all full-length RNAs of all classes, at single-base resolution, in an individual cell. Recently, several studies have reported single cell RNA sequencing analysis (51–55). We will discuss quantitative expression analysis, the detection of transcripts, and the identification of their splicing isoforms, using single-cell RNA sequencing in this section.

Several groups have demonstrated the application of single cell RNA sequencing in various biological systems (8, 51, 56–70) in recent years. For example, Tang et al. studied the blastomere cell using single cell RNA sequencing and found that 8–19% of the genes with multiple known transcript isoforms expressed at least two of those isoforms in the same blastomere cell (57). Ramsköld et al. applied Smart-Seq to study the gene expression profile of rare, circulating tumor cells from the blood of a melanoma patient, and found that the profile is highly correlated with those of melanoma cell lines, strongly indicating that the circulating cells originated from a melanoma tumor (15). Shalek et al. studied the transcriptomics of single immune cells and revealed a bimodality in expression and splicing (64).

The computational measurement of quantitative gene expression has been extensively studied in bulk cell sequencing analysis. Gene expression can be calculated from the number of sequencing reads mapped to a particular gene region. Current approaches in the analysis of quantitative gene expression include two steps: mapping RNA sequencing reads to gene regions, and calculating expression levels. In the first step, many tools have been developed for sequencing read mapping, such as TopHat (34), RUM (71), SpliceMap (72), MapSplice (73), GSNAP (74), BLAT (75), Bowtie (76), SOAP (77), and BWA (78). In the second step, RPKM (reads per kilobase per million reads) (79) and FPKM (fragments per kilobase per million fragments) (80) are commonly used to measure gene expression levels. Yan et al. used BWA to align reads to a reference genome (70) in a recent single cell RNA sequencing project. They selected genes with RPKM ≥ 0.1 for further analysis. Picelly et al. adopted STAR (81) to align sequence reads in another recent project, and then used RPKM for genes (82) to calculate RPKM. Shalek et al. used Tophat1 (34) to map reads to a reference genome, then used RSEM (83) to obtain the expression level of TPM (transcripts per million), and then used MISO (84) to locate the alternate splicing events (61).

The challenge of single cell RNA bioinformatics analysis is mainly due to the bias and distortion in the whole transcript amplification process. There are three major issues in single, whole transcript amplification: (1) amplification cannot generate full-length cDNAs; (2) transcripts are not amplified at the same ratio; and, (3) low abundant transcripts are difficult to detect.

It is usually difficult to get full-length transcripts in single cell RNA sequencing, and, therefore, 3′-end biases are often generated. Tang et al. only obtained RNA transcripts shorter than 3 kb in single mouse blastomere cells, missing 36% of the expressed genes (51). The median read coverage across expressed transcripts is 53.8% in the Quartz-Seq method, compared with 84.4% in conventional RNA sequencing (60). Ramsköld et al. demonstrated that the Smart-Seq technique can identify 40% of all full-length transcripts (15). The FPKM/RPKM values, which are commonly used to measure gene expression levels, do not consider bias across the transcripts, and therefore, may not suitable for single cell RNA sequencing in bioinformatics analysis. Furthermore, the pronounced 3′-end bias of whole transcript amplification may hamper the ability to identify alternate splicing differences in single cells.

GC content and cDNA length distribution may also induce artificial biases during whole transcript amplification. For example, Sasagawa et al. showed that unamplified isoforms from Quartz-Seq have a higher GC content, with value of 52.1%, versus the mean GC content of the amplified isoforms, with a value of 50.2%, which indicates that high GC content RNAs are difficult to amplify (60). They also found that amplified cDNAs have a longer length than the unamplified isoforms that correspond to the cDNA. Because of these reasons, we need to validate to what extent single-cell transcriptomes faithfully represent the RNA populations they reflect before amplification.

Bulk cell samples are usually sequenced at high depth to obtain low-abundance transcripts. However, in single cell sequencing, the low-abundance transcripts may not be so easily amplified. For example, the transcription detection rate is about 80% in Quartz-Seq and about 55% in Smart-Seq (60). Picelili found the observed variability between cells was mainly of a technical nature for low-abundance transcripts, whereas in medium- and high-abundance transcripts, variability between cells was mainly biological (58).

New methods are needed to generate an unbiased quantitative measure of transcript expression in single-cell transcriptomics analysis. Recently several new amplification protocols have been developed, but bias still exists to a certain extent. Based on our own experience, we suggest the following two potential solutions, which may help to address the problems of amplification bias and low coverage:

1. Consider a new standard expression level measurement beyond RPKM/FPKM in single cell RNA sequencing. Single cell RNA sequencing data usually has 3′ and/or 5′ biases; therefore, measuring expression levels using full-length transcripts may be inappropriate. One possible solution is to normalize expression levels by coverage lengths instead of by using full-length transcripts.
2. Reduce amplification biases by developing new bioinformatics approaches. We can systematically investigate how bias is generated during amplification to discover the patterns of bias. Machine learning (85–92) may be a powerful tool to study the distribution of bias and to predict amplification bias.

## Section III: Comparative Analysis of Single Cells

One important goal of single cell technology is to discover heterogeneity among cells (69, 93, 94). Dozens of single cells are usually sequenced in a single cell genomics project. The heterogeneity between cells can thus be found by comparative analysis between the single cells employed. Here we will describe the comparative analysis of single cells in the development and lineage structure of tumor and early embryonic development.

### Development and lineage structure of tumors

Genetic heterogeneity is very common in tumors, and is important information for reconstructing evolutionary history. This information may be averaged out in bulk cell sequencing (95). However, the comparative analysis of sequencing data from multiple single cells is a much more powerful technique for studying tumor population structure and evolution (18).

Navin et al. applied single-nucleus sequencing to investigate tumor population structure and evolution in two human breast cancer cases, and found that tumors grow by punctuated clonal expansions with few persistent intermediates (18). Hou et al. inferred tumor monoclonal origin in an essential thrombocythemia patient using single cell exome sequencing (96).

Although single cell sequencing can provide robust information regarding tumor heterogeneity and evolution, there are still several technical problems to resolve. The accuracy and sensitivity of detecting single cell variation can significantly affect single cell population analysis. In general, only mutations observed in multiple cells can confidently be considered real mutations. As a result, some rare mutations or cell clones may not be able to be identified with a reasonable confidence level. Another question is how many cells should be sequenced in a single cell sequencing project? The sequencing cost of a single cell is still relatively high, and we need a statistic model to evaluate the appropriate number of single cells in a project.

### Embryonic development

Single cell RNA sequencing can provide insight into the dynamic expression of key genes to explore the relationship among the different stages of stem cells (57). Tang et al. traced the derivation of embryonic stem cells from the inner cell mass by single-cell RNA sequencing analysis (97), providing insight into the dynamic molecular changes that accompany cell fate changes based on the expression of both mRNA and microRNA. Xue et al. reported a comprehensive analysis of transcriptome dynamics from oocyte to morula in both human and mouse embryos, and identified embryonic genome activation events (8). These results provide valuable resources for dissecting gene regulatory mechanisms, and for understanding the underlying progressive development of early mammalian embryos. For example, single cell RNA sequencing has a great potential for discovering previously unrecognized biological distinctions between two-cell, four-cell, eight-cell, and later stages of embryogenesis. However, the high level of noise inherent in single cell genomics is hard to address, because of technical limitations in both experimental preparations and computational approaches, due to biological reasons and the limited amount of input material available. Future studies based on hundreds or thousands of single cells with new bioinformatics approaches will enable analyses to reconstruct intracellular genetic circuits, enumerate and redefine cell developmental states and types, and understand cellular decision-making on a genomic scale.

## Conclusion

Single cell genomics analysis not only provides a more precise measurement, but is also a decisive move toward a fundamental understanding of the biology of cells. The ever-increasing power of DNA sequencing technology means that it will soon be possible to sequence every nucleic acid in many thousands of cells.

At present, single cell sequencing techniques still have two major shortcomings: low genome coverage, and high amplification bias. Despite the limitations, these still-evolving technologies will eventually revolutionize research in oncology, neuroscience, cell development, and microbiology. Partly through innovations in microfluidics (98, 99) and next generation sequencing technologies, we expect that the primary nucleic acid sequence analysis of single cell genomic DNAs and RNAs will be solved in a few years.

However, few existing bioinformatics software packages exist for the purpose of single cell genomics data analysis. Continued advances in the application of singe cell sequencing technologies in biological research will require development of new algorithms and software able to handle the specific characteristics of these technologies. In particular, we need tools to evaluate the performance of different single cell sequencing technologies. Technical standards should be built to evaluate the genome coverage and amplification biases, so that the results from different technologies can be compared with each other. Meanwhile, new tools are needed to manage the large amounts of data generated by single cell sequencing technologies, which is expected to be one order magnitude larger than regular bulk sequencing projects. An open-source and shared model will accelerate the progress by allowing the scientific community to join forces in addressing the challenges and promises of the new technologies.

## Conflict of Interest Statement

The authors declare that the research was conducted in the absence of any commercial or financial relationships that could be construed as a potential conflict of interest.
